# Studies on *Lygus pratensis*’ (Hemiptera: Miridae) Flight Ability

**DOI:** 10.3390/insects15100762

**Published:** 2024-09-30

**Authors:** Yixiang Zheng, Pengfei Li, Tailong Li, Kunyan Wang, Changqing Gou, Hongzu Feng

**Affiliations:** 1 Agricultural College, Tarim University, Alar 843300, China; 10757222015@stumail.taru.edu.cn (Y.Z.); 18167525229@163.com (P.L.);; 2 Key Laboratory of Integrated Pest Management (IPM) of Xinjiang Production and Construction Corps in Southern Xinjiang, Alar 843300, China; 3 Scientific Observing and Experimental Station of Crop Pests in Alar, Ministry of Agriculture, Alar 843300, China; 4 The National-Local Joint Engineering Laboratory of High Efficiency and Superior-Quality Cultivation and Fruit Deep Processing Technology on Characteristic Fruit Trees, Alar 843300, China

**Keywords:** mirid bug, flight capacity, environmental, individual development, flight milling

## Abstract

**Simple Summary:**

*Lygus pratensis* transfers frequently between cotton and different host plants, harms a wide range of crops, possesses a strong dispersal ability, and poses great difficulty in predicting pests, but its flight ability and the factors that affect it are not well understood. We found that *Lygus pratensis* possesses strong flight ability, that the strength of flight ability is affected by the temperature and relative humidity of the environment, and that an unsuitable environment will greatly reduce the flight ability. Furthermore, we found that flight ability will be enhanced and then weakened with the increase in age, that the flight ability of females is slightly stronger than that of males, and that mating will increase the flight ability of females and weaken the flight ability of males. The above results can provide a basis for predicting the spreading range of *Lygus pratensis* in different seasons or different reproductive periods.

**Abstract:**

*Lygus pratensis* (Linnaeus) is an important agricultural pest with a strong ability to move and spread between hosts. However, *L. pratensis*’ flight potential and factors affecting its flight ability are unclear. We used the insect flight information system (flight mill) to determine the effects of temperature, humidity, age, sex, and mating on *L. pratensis*’ flight ability in an artificial climate chamber. Temperature and relative humidity significantly affected *L. pratensis*’ flight ability; however, low and high temperature, as well as low humidity, were unsuitable, and the optimal flight environment was 20–28 °C and 60–75% RH. *Lygus pratensis*’ flying ability initially increased and then decreased with age and was highest at 10 days old (flight rate: 71.43%; total flight distance: 18.63 ± 1.89 km; total flight time: 6.84 ± 0.60 h). At 15 days old, flight speed was the highest (3.36 ± 0.18 km h^−1^). Sex had little effect on *L. pratensis*’ flying ability; it was marginally stronger for females than males, but the difference was insignificant. Mating increased female flying ability but decreased that of males, but the difference was insignificant. Overall, *L. pratensis* had strong flight dispersal ability, was largely unaffected by sex and mating, and optimal flight conditions were mild temperature and humidity. This knowledge provides a scientific basis for *L. pratensis* outbreak prediction, prevention, and control.

## 1. Introduction

The plant bug *Lygus pratensis* (Linnaeus) (Hemiptera: Miridae) is widely distributed in Eurasia, North America, and northern Africa [1] and is an important agricultural pest in Xinjiang, China, where it causes cotton boll shedding. Recently, *L. pratensis* has again become rampant, with boll damage in cotton fields as high as 56% [2,3]. Simultaneously, *L. pratensis* can invade orchards and damage flower buds and young fruits. Fruit trees such as fragrant pear, apple, jujube, and walnut may suffer fruit deformity in light cases, and numerous flowers and fruits may fall off in severe cases [4,5]. There are many *L. pratensis* host plant species [6], but most of them are annual crops and weeds, which are suitable for feeding and rapid reproduction. Therefore, inter-host diffusion and transfer is an important link in the life history of *L. pratensis*. Overwintering *L. pratensis* adults first feed and reproduce in early spring in wheat fields, alfalfa fields, and weeds in orchards. Then, there are several large migration activities: first-generation adults move to cotton fields in mid-June, and then in early September, and third-generation adult insects begin to move from the cotton fields to autumn hosts (Lepidaceae and Asteraceae weeds) [7,8]. In *L. pratensis,* there is an important relationship between its ability to move and spread between hosts and its flying ability.

*Miridae* have strong migration and diffusion ability and readily proliferate when in a suitable living environment [9]. *Lygus lineolaris* (Palisot de Beauvois) and *Lygus rugulipennis* (Poppius) were captured in the mid-20th century at altitudes of 1500 m and 900 m [10,11]. Carrière et al., using GIS technology, monitored *Lygus hesperus* (Knight) and found that it had a strong flight diffusion ability in host–plant transfer [12]. Indoor flight tests (flight grinding and vertical flight capsule) on *L. lineolaris* and *L. hesperus*, respectively, identified strong flight potential [13,14]. Lu was the first to study mirid flight capabilities in China and found that *Apolygus lucorum* (Meyer-Dür), *Adelphocoris suturalis* (Jakovlev), *Adelphocoris fasciaticollis* (Reuter), and *Adelphocoris lineolatus* (Goez) had strong flying ability [15]. Therefore, it is speculated that *L. pratensis* also has a strong flight ability, but no studies have yet been conducted.

The insect flight ability test is an important method for studying insect flight laws [16]. The indoor test primarily uses an insect flight law simulation device, usually called a flight mill. Insects are fixed on the flight mill to make them fly autonomously; then, relevant insect flight parameters are obtained through computer analysis and calculation [17,18].

Many factors affect insect flying ability, including external environmental factors and internal factors (age, sex, reproduction). After insect emergence, flight muscles develop gradually, relevant enzymes and energy substances in the body are constantly supplemented, and flight ability enhanced with increasing age. Simultaneously, insects consume considerable energy for reproductive activities, which will also affect flight ability [10,19,20]. There are also some differences in flying ability between male and female insects. Most female insects have stronger flight ability than males, which is conducive to finding a wider range of host plants for reproduction and egg laying and may be related to sex size differences [21,22]. Food, temperature, humidity, and light intensity are also critical to flying ability [19,23]. Temperature and humidity play a key role in insect growth and development and also affect insect flight ability. Low (high) temperature and low humidity will lower insect body water content, which will reduce metabolic capacity and material conversion ability, thus weakening flight ability [24,25].

In this study, the effects of temperature and humidity, age, sex, and mating on *L. pratensis* flight ability were determined to explore the ability of *L. pratensis* to move and spread in the field and to provide a scientific basis for forecasting and control.

## 2. Materials and Methods

### 2.1. Study Insects

Female and male *L*. *pratensis* were captured in an alfalfa field in Aral City (Xinjiang, China) (40°52′15″ N, 81°30′94″ E) in April 2023, raised at 24 °C with a 5:9 (L:D) photoperiod, relative humidity of 70 ± 10%, and light intensity of 480 ± 20 lx, and fed cauliflower. Females and males of *L*. *pratensis* were raised together to allow for mating and the production of first- and second-generation adults.

### 2.2. Test Instruments and Materials

The test instrument consisted of an RXZ intelligent artificial climate box (Ningbo Jiangnan Instrument Factory, Ningbo, China), a JIADUO insect flight information System (Hebi Jiaduo Weinong Agriculture and Forestry Technology Co., Ltd., Hebi, China), a flying mill (FXM-Z, 24-way), an insect small flying mill (FXM-X, 12-way), and a Leica M205C Stereo Microscope (Leica Biosystems, Nußloch, Germany).

The test material included a CO_2_ generator (reaction equation); a flight power transmission unit made of copper wire, insect needles, and black blackout paper, an insect-box (made by us); and 502 glue (Deli Group Co., Ltd., Ningbo, China).

### 2.3. Tethered Flight Test

Before the test, *L. pratensis* were treated with CO_2_ for 25 s to induce coma; then, the tip of the boom of the flight power transmission unit was attached to the *L. pratensis* nolum with 502 glue. After *L. pratensis* awakened, the flight power transmission device was placed between the two insect small flying mill magnets. Paying attention to the black blackout paper suspended in the middle of the infrared counting sensor recess, the small flying mill was then placed into the RXZ intelligent artificial climate chamber for testing. The test began at 9:00 AM and lasted for 24 h. Flight distance, time, and average flight speed over the 24 h period were recorded by the insect flight information system computer. *Lygus pratensis* death or flight times less than 10 min were not included in the statistics. Insects with total flight time greater than 10 min were deemed fliers and included in the data, while those that did not (non-fliers) were not included [13].

### 2.4. L. pratensis’ Flight Ability at Different Temperatures

After emergence, female insects were raised alone until 10 days old and then fixed on the flight mill and put into the artificial climate chamber for flight tests. The artificial climate chamber was set at a photocycle of 15:9 (L:D), light intensity 480 ± 20 lx, relative humidity 75%, and temperature gradients of 16, 20, 24, 28, 32, and 36 °C, successively. There were 6 temperature treatments with 20 repetitions for each treatment (20 worms).

### 2.5. L. pratensis’ Flight Ability under Different Relative Humidities

After emergence, female insects were raised alone until 10 days old and then fixed on the flight mill and put into the artificial climate chamber for flight tests. The artificial climate chamber were set at a photocycle of 15:9 (L:D), light intensity 480 ± 20 lx, temperature 24 °C, and relative humidity 30, 45, 60, 75, and 90%, successively. There were 5 humidity treatments with 20 repetitions for each treatment (20 worms).

### 2.6. L. pratensis’ Flight Ability at Different Ages

Female *L. pratensis* that emerged on the same day were kept alone without mating and tested for flight ability at different ages (1, 5, 10, 15, 20, 25, and 30 d old). There were 7-day-old treatments with 20 replicates per treatment (20 worms). Flight parameters were measured, and flight probability was calculated (fliers). Test conditions: temperature: 24 °C; photoperiod: 15:9 (L:D); relative humidity: 70 ± 10%; light intensity: (480 ± 20) lx.

### 2.7. L. pratensis’ Flight Ability in Relation to Sex and Mating

Male and female adult *L. pratensis* were reared separately without mating. The flight ability of unmated females and males was tested on the 7th day of breeding. In another group, male and female *L. pratensis* adults were bred in pairs. According to Lu and proof of pre-test, breeding females and twice the number of males together can ensure female mating, and the same treatment was applied to mated females and mated males [15]. The flight ability of mated females and males was tested on the 7th day of breeding. *Lygus pratensis* ontogeny studies show that it begins to lay eggs around 8 days after emergence [26]. To avoid the influence of spawning on the test, the flight ability test was started on the 7th day of emergence. The experiment was divided into 4 treatments—unmated females, unmated males, mated females, and mated males—with 20 replicates per treatment (20 worms).

Measurement of wing area, body weight, and body length of 10 day old *Lygus pratensis* male and female adults. Five adults of each sex were taken, the anterior and posterior wings of one side were separated, the wings were photographed using a Leica M205C stereomicroscope, the photographs of each wing were processed using ImageJ (v1.54d), and the anterior and posterior wing areas were measured. A group of 10 adults of each sex was taken for weighing and the weight of one *L. pratensis* was calculated; each group was repeated three times, and another 10 adults of each sex were taken for body length measurement.

### 2.8. Data Analysis

Total flight distance, total flight time, and average flight speed data were analyzed using SPSS 25.0. A normality test was performed on the raw data first. If the data did not conform to a normal distribution, logarithmic conversion was performed, and then the Duncan multiple range test (new complex range method) was used to compare differences (*p* < 0.05). *t*-tests were used to compare the effects of sex and mating on *L. pratensis*’ flight ability (*p* < 0.05).

The relationship between temperature and relative humidity and total flight distance, total flight time, and average flight speed was expressed as a curvilinear equation. In the model, Y was the conversion value (LgX) of the total flight distance (or time, speed) and X represented the variables (temperature, relative humidity).

## 3. Results

### 3.1. The Effect of Temperature on Flight Ability

**Figure 1:**
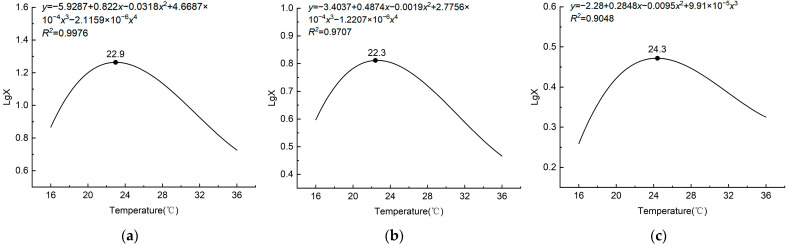
Nonlinear curve fitting of temperature versus flight distance (**a**), flight time (**b**), and flight speed (**c**). LgX is the logarithmic value of the flight distance (time, speed). ● is the temperature value of the maximum flight distance (time, speed).

Temperature significantly affected *L. pratensis*’ total flight distance (*F* = 9.747; df = 5, 144; *p* < 0.05), which initially increased and then decreased with increasing temperature. At 24 °C, *L. pratensis*’ total flight distance was 18.63 ± 1.89 km, which was not significantly different from at 20 °C (15.67 ± 2.20 km); however, it was significantly higher than at other temperatures. At 28 °C, it was 13.14 ± 1.94 km; at 32 °C, 8.62 ± 1.30 km; and the shortest flight distance was at 36 °C (5.30 ± 1.03 km) (Table 1). According to the curve model, *L. pratensis*’ flight velocity reached its maximum at 22.9 °C (Figure 1a).

Temperature significantly affected *L. pratensis*’ total flight time (*F* = 4.845; df = 5, 144; *p* < 0.05), which initially increased and then decreased with increasing temperature. At 24 °C, *L. pratensis*’ total flight time was the longest (6.84 ± 0.60 h), which was higher (although not significantly) than that at 20 °C (5.86 ± 0.79 h). Total flight time at 16 °C was similar to that at 28, 32, and 36 °C (Table 1). According to the curve model, *L. pratensis*’ flight time reached its maximum at 22.3 °C (Figure 1b).

There was a significant effect of temperature on the average flight speed of *L. pratensis* (*F* = 3.543; df = 5, 144; *p* < 0.05), with a trend of increasing and then decreasing flight speeds with increasing temperature. *Lygus pratensis* had the fastest mean flight speed at 24 °C (2.84 ± 0.27 km h^−^^1^), which was not significantly different from the mean flight speeds at 20 °C, 28 °C, and 32 °C but was significantly different from the lowest temperature of 16 °C (1.79 ± 0.15 km h^−^^1^) and the highest temperature of 36 °C (2.09 ± 0.21 km h^−^^1^) (Table 1). According to the curve model, *L. pratensis*’ flight velocity reached its maximum at 24.3 °C (Figure 1c).

### 3.2. Effect of Relative Humidity on Flight Ability

**Figure 2:**
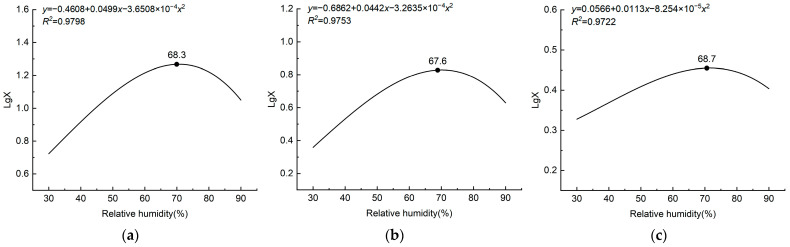
Nonlinear curve fitting of relative humidity to flight distance (**a**), flight time (**b**), and flight speed (**c**). LgX is the logarithmic value of the flight distance (time, speed). ● is the relative humidity value of the maximum flight distance (time, speed).

Relative humidity significantly affected *L. pratensis*’ total flight distance (*F* = 9.086; df = 4, 95; *p* < 0.05), which initially increased and then decreased with increasing relative humidity. *Lygus pratensis* had the longest total flight distance at 75% RH (18.63 ± 1.89 km), which was not significantly different from that at 60% RH (15.75 ± 2.17 km) but significantly higher than at other relative humidities. There was no significant difference in total flight distance between 90% relative humidity (11.14 ± 1.69 km) and 60% relative humidity (15.75 ± 2.17 km). When the minimum relative humidity was 30%, the total flight distance was 5.25 ± 1.03 km, which was significantly lower than at other relative humidities (Table 2). According to the curve model, *L. pratensis*’ total flight distance reached a maximum when relative humidity was 68.3% (Figure 2a).

*Lygus pratensis*’ total flight time was significantly affected by relative humidity (*F* = 9.664; df = 4, 95; *p* < 0.05), which initially increased and then decreased with increasing humidity. When relative humidity was 75%, total flight time was the longest (6.84 ± 0.60 h), and the second-longest total flight time (5.79 ± 0.73 h) was not significantly different. Total flight times at 45 and 90% relative humidity were similar (4.23 ± 0.54 h and 4.22 ± 0.52 h, respectively) but significantly lower than total flight time at 75% relative humidity. At 30% relative humidity, *L. pratensis* had the shortest total flight time (2.26 ± 0.33 h), which was significantly lower than at other relative humidities (Table 2). According to the curve model, *L. pratensis*’ total flight time reached its maximum at 67.6% relative humidity (Figure 2b).

*Lygus pratensis*’ average flight speed was not significantly affected by relative humidity (*F* = 1.302; df = 4, 95; *p* = 0.275), which initially increased and then decreased with increasing relative humidity. *L. pratensis* had the highest average flight speed (2.84 ± 0.270 km h^−^^1^) at 75% relative humidity and the slowest (2.13 ± 0.27 km h^−^^1^) at 30% relative humidity, but the average flight speed difference was not significantly different (Table 2). According to the curve model, *L. pratensis*’ average flight speed reached its maximum at 68.7% relative humidity (Figure 2c).

### 3.3. The Influence of Age on Flight Ability

*Lygus pratensis*’ flight ability initially increased and then decreased with increasing age. Total flight distance (*F* = 15.915; df = 6, 135; *p* < 0.05), total flight time (*F* = 10.363; df = 6, 133; *p* < 0.05) and average flight speed (*F* = 7.822; df = 6, 147; *p* < 0.05) were significantly affected by age. *Lygus pratensis* had the weakest flight ability at 1 day old, with the lowest flight rate (30.95%), total flight distance (3.97 ± 0.68 km), and total flight time (2.10 ± 0.41 h), and the slowest average flight speed (2.19 ± 0.11 km) at 5 days old. However, flight abilities at 1, 25, and 30 days old were similar to at 5 days old and not significantly different. At 10 days old, *L. pratensis* had the highest flight rate (71.43%), total flight distance (18.63 ± 1.89 km), and total flight time (6.84 ± 0.60 h). However, average flight speed was fastest at 15 days old (3.36 ± 0.18 km h^−^^1^). There was no significant difference between total flight distance and average flight speed at 10, 15, and 20 days old; total flight time did not differ between 10 and 15 days old; and flight ability at 10 days old was significantly higher than at 1, 5, 25, and 30 days old (Table 3).

### 3.4. The Effect of Sex and Mating on Flight Ability

In the mating state, female *L. pratensis*’ flight ability (19.54 ± 2.09 km, 6.95 ± 0.70 h, 2.94 ± 0.20 km h^−^^1^) was stronger than male (10.17 ± 1.50 km, 4.50 ± 0.62 h, 2.52 ± 0.23 km h^−^^1^), and there were significant differences in total flight distance (*t* = 3.647; df = 38; *p* < 0.05) and total flight time (*t* = 2.628; df = 38; *p* < 0.05). Average flight speed (*t* = 1.418; df = 38; *p* = 0.1655) was not significantly different. However, unmated female *L. pratensis*’ flight ability (15.86 ± 1.96 km, 6.13 ± 0.74 h, 2.68 ± 0.20 km h^−^^1^) was also stronger than unmated male (12.92 ± 1.72 km, 4.88 ± 0.54 h, 2.56 ± 0.21 km h^−^^1^). But differences in total flight distance (*t* = 1.130; df = 38; *p* = 0.266), total flight time (*t* = 1.370; df = 38; *p* = 0.179), and average flight speed (*t* = 0.412; df = 38; *p* = 0.682) were not significant (Table 4).

*Lygus pratensis* mated female flight ability was better than for non-mated females, but their total flight distance (*t* = 1.287; df = 38; *p* = 0.206), total flight time (*t* = 0.806; df = 38; *p* = 0.425), and average flight speed (*t* = 0.937; df = 38; *p* = 0.355) were not significantly different. *Lygus pratensis* mated male flight ability was weaker than for non-mating males, but the differences in total flight distance (*t* = 1.204; df = 38; *p* = 0.236), total flight time (*t* = 0.464; df = 38; *p* = 0.645), and average flight speed (*t* = 0.145; df = 38; *p* = 0.885) were not significant (Table 5).

There was no significant difference in forewing area (*t* = 0.372; df = 8; *p* = 0.719) between the females and males, nor was there a significant difference in hindwing area (*t* = 0.291; df = 8; *p* = 0.778). The body weight (8.09 ± 0.13 mg) and body length (5.25 ± 0.07 mm) of females were significantly greater than those of males (*t* = 13.531; df = 4; *p* < 0.05) (*t* = 4.794; df = 18; *p* < 0.05) (Table 6).

## 4. Discussion

*Lygus pratensis*’ flight ability was significantly affected by temperature and relative humidity in the flight environment. *Lygus pratensis*’ flight ability was highest when the temperature was 24 °C and the relative humidity was 75%, and the total flight distance was 18.63 ± 1.20 km. Total flight time was 6.84 ± 0.60 h, and average flight speed was 2.84 ± 0.27 km h^−1^. According to the curve model, *L. pratensis’* flight ability initially increased and then decreased with increasing temperature and relative humidity, and optimal flight temperature and relative humidity were 20–28 °C and 65–70%, respectively. *L. pratensis*’ flight ability decreased significantly when the temperature was lower than 20 °C or higher than 28 °C. *Lygus pratensis*’ total flight distance and total flight time were shortest when the temperature was 16, 32, and 36 °C, respectively, indicating that the optimum temperature range suitable for *L. pratensis*’ flight was 20–28 °C, which is consistent with the biological characteristics of *L. pratensis* [27,28]. In an average velocity comparison, only the lowest and the highest temperature affected *L. pratensis’* flight velocity; all other temperatures had no significant effect. *L. pratensis*’ flight ability was strongest when relative humidity was 60–75% and was weakened when the air humidity was outside this range. Field investigation showed that relative humidity drives *L. pratensis* population changes, a feature also noted by Bai et al. [29]. *Lygus pratensis* had the weakest flight ability at low (16 °C) and high temperature (36 °C) and low humidity (30% RH). It is hypothesized that the above results reflect the insects’ ability to regulate their own temperature by evaporating water from their bodies in high temperatures [24], as well as in low humidity environments, lowering the likelihood that unsuitable temperatures and humidity will cause dehydration, thus reducing energy transformation [25] and the ability to fly. Previous studies on *Carpomya vesuviana* (Costa) (Diptera: Tephritidae) found that high- and low-temperature environments reduced the utilization of flight energy substances in the body, thus reducing flight ability [30]. Optimum conditions for *L. pratensis* are similar to *A. lucorum*, *A. suturalis*, *A. fasciaticollis*, and *A. lineolatus* optimal flight environments (temperature 18–23 °C, relative humidity 64–68%) [15]. They are also consistent with temperature and humidity effects on optimal flight in other insect studies; for example, *Spodoptera frugiperda*’s (J. E. Smith) (Lepidoptera: Noctuidae) optimal flight temperature and humidity were 20–25 °C and 60–90% RH [31], while *Agrypon flexorius* (Thunberg) (Hymenoptera: Ichneumonidae) had the highest flight capability at 20 °C and 60% RH [32]. *Sitophilus zeamais*’ (Motschulsky) (Coleoptera: Curculionidae) flight ability decreased significantly at 34 °C [33], whereas *Mamestra brassicae* (Linnaeus) (Lepidoptera: Noctuidae) had the best flight capability from 23–25 °C and 64–75% RH [34].

According to the age-related flight ability test results, *L. pratensis* had the longest total flight distance (18.63 ± 1.89 km) and total flight time (6.84 ± 0.60 h) at 10 days old and the highest average flight speed (3.36 ± 0.18 km h^−1^) at 15 days old. *Apolygus lucorum* and other species of mirids are more capable of flying than *L. pratensis.* For example, *A. suturalis’* flight ability (flight distance of 40 km, flight time of 7.5 h, flight speed of 5.25 km h^−1^) was significantly better than that of *L. pratensis.* Only *A. lineolatus* is close to *L. pratensis* in flight ability, while *A. lucorum* flew 96.55% of the time at 10 days old, about 25% higher than the grass bug [15]. However, some bugs, such as *L. lineolaris*, had a flight rate of 42% [13], and *L. hesperus* had a flight rate of only about 20% [14], both significantly lower than *L. pratensis*. *Lygus pratensis*’ flight ability initially increased and then decreased with age. *Lygus pratensis*’ flight ability was weakest at 1 day old, strongest at 10 days old, declined slowly at 15–20 days of age, and then decreased sharply after 25 days old. This pattern is similar to other insects such as *Corythucha ciliata* (Say) (Hemiptera: Tingidae) [35] and *Agrotis segetum* (Denis and Schiffermulle) (Lepidoptera: Noctuidae) [36], in addition to other mirids. According to previous studies and analyses, insects that have just emerged (1 day old) have imperfect flight muscle development, and the enzymes and energy substances required for flight are insufficient, so flight ability is relatively weak. *Lygus pratensis* began to lay eggs at 8 days old, which may have affected flight by transferring more energy to reproduction. When *L. pratensis* nears the end of life after 30 days, energy materials in the body decline and degradation of flight organs begins [10,37].

Both female and male *L. pratensis* have strong flight ability. Unmated adult (7 days old) females (15.86 ± 1.96 km, 6.13 ± 0.74 h, 2.68 ± 0.20 km h^−1^) had stronger flight ability than unmated males (12.92 ± 1.72 km, 4.88 ± 0.54 h, 2.56 ± 0.21 km h^−1^), although the difference was not significant. This is similar to *A. suturalis*, *A. fasciaticollis*, and *A. lineolatus* [15]. The study of wing area, body weight, and body length of *L. pratensis* revealed that females and males had similar wing areas with no significant differences, but females of the same day old had larger body weights and body lengths than males, and there were physiological and morphological differences, which might be the main reason for the females’ stronger flight ability when compared to the males [21,38]. Total flight distances and times of female *L. pratensis* after mating were significantly higher than male *L. pratensis*. Experiments on the effects of mating on *L. pratensis*’ flight ability showed that mating enhanced female flight ability and weakened that of the male, although the differences were not significant. This may be the main reason for the superior female flight ability after mating. Therefore, there is a small difference in flight ability between female and male *L. pratensis*, but it is more significant after mating. Previous studies have shown that other female and male insects have different flight abilities. For example, for *C. vesuviana* [30] and *Dastarcus helophoroides* (Fairmaire) (Coleoptera: Bothrideridae) [39], female and male insects did not differ in flight ability; but for *L. hesperus* [40] and *A. lucorum* [21], females flew better than males. Conversely, the male *Spissistilus festinus*’ (Say) (Hemiptera: Membracidae) flight distance was greater than that of the female [41]. Mating also has different effects on insect flight ability. In some insects, after mating, females will enhance their flight ability to find new host plants for laying eggs [20]. For example, the flight distance and flight time of mated *Amyelois transitella* (Walker) (Lepidoptera: Pyralidae) females were significantly higher than unmated females [42]. Conversely, after mating, for *Euzophera pyriella* (Yang) (Lepidoptera: Pyralidae) [43] and *A. segetum* [36], female flight ability decreased and was lower than in the non-mating state. This phenomenon may be caused by mating activities, stimulating the transfer of energy substances to reproduction and inhibiting flight muscles’ development [44].

The flight distance, time, and speed of *L. pratensis* were measured in the laboratory, and its theoretical maximum dispersal distance for a single day (24 h) was approximately 18 km, but this was not free flight. Flight mills could visually compare the effects of different factors on the strength of an insect’s flight [45,46], but there were some differences from free flight; for example, in a comparison of flight mills and free-flight chambers, *Drosophila suzukii* (Matsumura) (Diptera: Drosophilidae) was found to fly longer in free-flight chambers [47]. *Agrilus planipennis* (Fairmaire) (Coleoptera: Buprestidae) was three times as fast in free flight as it was on the flight mill [48]. Thus, the flight experiments in laboratory might over- or under-estimate the flight potentials of insects [49,50]. The flight tests in the laboratory could only be used as predictors of the potential dispersal capacity of insects in the field and need to be combined with outdoor monitoring methods to predict the extent of *L. pratensis* dispersal more accurately. The effects of temperature, humidity, age, sex, and mating on *L. pratensis*’ flight ability were determined. Since flight ability was tested under changes in a single environmental factor, our research results only reflect the effects of these changes and do not accurately reflect *L. pratensis*’ flight ability with temperature and humidity changes during the day. In the future, *L. pratensis’* flight ability can be tested in the natural environment to investigate its relationship with environmental factors further. Our experimental results preliminarily proved that mating had little effect on *L. pratensis*, but whether it had “Oogenesis-flight syndrome” remains to be determined.

## 5. Conclusions

Temperature and humidity significantly affect *L. pratensis*’ flight ability. Low temperature, high temperature, and low humidity are unsuitable for flight, and the optimal flight environment is 20–28 °C and 60–75% RH. *Lygus pratensis*’ flight ability initially increased and then decreased with age and was strongest at 10 days old. Sex had little effect on flying ability; female flying ability was slightly stronger than males, but the difference was in significant. Mating enhanced female *L. pratensis*’ flight ability and weakened that of males, but the differences were insignificant.

## Figures and Tables

**Table 1:** Effects of different temperatures on flight ability of *L. pratensis*.

Temperature (°C)	Total Flight Distance (km)	LgX	Total Flight Time (h)	LgX	Average Flying Speed (km h^−1^)	LgX
16	7.35 ± 1.31 d	0.867	3.98 ± 0.64 bc	0.600	1.79 ± 0.15 c	0.252
20	15.67 ± 2.20 ab	1.195	5.86 ± 0.79 ab	0.768	2.78 ± 0.26 ab	0.444
24	18.63 ± 1.89 a	1.270	6.84 ± 0.60 a	0.835	2.84 ± 0.27 a	0.453
28	13.14 ± 1.94 bc	1.118	4.86 ± 0.63 bc	0.687	2.75 ± 0.20 ab	0.439
32	8.62 ± 1.30 cd	0.936	4.04 ± 0.68 bc	0.606	2.53 ± 0.26 ab	0.402
36	5.30 ± 1.03 d	0.724	2.905 ± 0.523 c	0.463	2.085 ± 0.213 bc	0.319

The data in the table are average ± SE, and different lowercase letters indicate significant differences in Duncan’s multiple comparisons (*p* < 0.05).

**Table 2:** Effects of different relative humidity values on flight ability of *L. pratensis*.

Relative Humidity (%)	Total Flight Distance (km)	LgX	Total Flight Time (h)	LgX	Average Flying Speed (km h^−1^)	LgX
30	5.25 ± 1.03 d	0.720	2.26 ± 0.33 c	0.355	2.13 ± 0.27 a	0.328
45	10.46 ± 1.57 c	1.020	4.23 ± 0.54 b	0.627	2.45 ± 0.23 a	0.389
60	15.75 ± 2.17 ab	1.197	5.79 ± 0.73 ab	0.763	2.76 ± 0.25 a	0.441
75	18.63 ± 1.89 a	1.270	6.84 ± 0.60 a	0.835	2.84 ± 0.27 a	0.453
90	11.14 ± 1.69 bc	1.047	4.22 ± 0.52 b	0.625	2.54 ± 0.21 a	0.404

The data in the table are average ± SE, and different lowercase letters indicate significant differences in Duncan’s multiple comparisons (*p* < 0.05).

**Table 3:** Effects of different ages on flight ability of *L. pratensis*.

Day of Age (d)	Flight Probability (%)	Total Flight Distance (km)	Total Flight Time (h)	Average Flying Speed (km h^−1^)
1	30.95	3.97 ± 0.68 d	2.10 ± 0.41 d	2.28 ± 0.17 c
5	64.29	9.62 ± 1.42 b	4.32 ± 0.54 bc	2.19 ± 0.11 c
10	71.43	18.63 ± 1.89 a	6.84 ± 0.60 a	2.84 ± 0.16 ab
15	69.05	17.32 ± 2.06 a	5.45 ± 0.73 ab	3.36 ± 0.18 a
20	57.14	14.79 ± 1.87 a	4.68 ± 0.58 bc	3.19 ± 0.16 a
25	40.47	8.65 ± 1.21 bc	3.32 ± 0.48 cd	2.60 ± 0.18 bc
30	38.09	4.54 ± 0.85 cd	2.14 ± 0.36 d	2.22 ± 0.18 c

The data in the table are average ± SE, and different lowercase letters indicate significant differences in Duncan’s multiple comparisons (*p* < 0.05).

**Table 4:** Effect of sex on flight ability of *L. pratensis*.

Mating Status	Sex	Day of Age (d)	Total Flight Distance (km)	Total Flight Time (h)	Average Flying Speed (km h^−1^)
Mated	Female	7	19.54 ± 2.09 a	6.95 ± 0.70 a	2.94 ± 0.20 a
	Male	7	10.17 ± 1.50 b	4.50 ± 0.62 b	2.52 ± 0.23 a
Unmated	Female	7	15.86 ± 1.96 a	6.13 ± 0.74 a	2.68 ± 0.20 a
	Male	7	12.92 ± 1.72 a	4.88 ± 0.54 a	2.56 ± 0.21 a

The data in the table are average ± SE, and different lowercase letters indicate significant differences in *t*-test comparisons (*p* < 0.05).

**Table 5:** The effect of mating on the flight ability of *L. pratensis*.

Sex	Mating Status	Day of Age (d)	Total Flight Distance (km)	Total Flight Time (h)	Average Flying Speed (km h^−1^)
Female	Mated	7	19.54 ± 2.09 a	6.95 ± 0.70 a	2.94 ± 0.20 a
	Unmated	7	15.86 ± 1.96 a	6.13 ± 0.74 a	2.68 ± 0.20 a
Male	Mated	7	10.17 ± 1.50 a	4.50 ± 0.62 a	2.52 ± 0.23 a
	Unmated	7	12.92 ± 1.72 a	4.88 ± 0.54 a	2.56 ± 0.21 a

The data in the table are average ± SE, and different lowercase letters indicate significant differences in *t*-test comparisons (*p* < 0.05).

**Table 6:** Comparison of wing area and body size of adult *L. pratensis* males and females.

Sex	Day of Age (d)	Forewing Area (mm^2^)	Hindwing Area (mm^2^)	Weight (mg)	Body Length (mm)
Female	10	5.70 ± 0.22 a	5.86 ± 0.40 a	8.09 ± 0.13 a	5.25 ± 0.07 a
Male	10	5.89 ± 0.44 a	5.71 ± 0.33 a	5.62 ± 0.13 b	4.71 ± 0.09 b

The data in the table are average ± SE, and different lowercase letters indicate significant differences in *t*-test comparisons (*p* < 0.05).

## Data Availability

The data presented in this study are available on request from the corresponding author.
